# Perception of Food Safety Associated with Entomophagy among Higher-Education Students: Exploring Insects as a Novel Food Source

**DOI:** 10.3390/foods12244427

**Published:** 2023-12-10

**Authors:** Fernando Cantalapiedra, Ana Juan-García, Cristina Juan

**Affiliations:** 1 Laboratory of Food Chemistry and Toxicology, Faculty of Pharmacy, University of Valencia, Av. Vicent Andrés Estellés s/n, Burjassot, 46100 Valencia, Spain; fernando.cantalapiedra@uv.es (F.C.); cristina.juan@uv.es (C.J.); 2 Veterinary Area of Public Health Center (CSP) Manises (Conselleria Sanitat-Generalitat Valenciana), C. Ceramista Alfons Blat s/n, Manises, 46940 Valencia, Spain

**Keywords:** novel food, risk perception, valorization, insects, food safety

## Abstract

Edible insects can diversify diets, improve livelihoods, contribute to food and nutrition security, and have a smaller ecological impact. The European Union has categorized insects as novel food, and recently, in 2021 and 2022, two species, *Tenebrio molitor* and *Acheta domesticus*, were authorized for commercialization. The acceptance and perception of food risk derived from insect consumption vary depending on factors impacting insect consumption acceptability, including neophobic tendencies, gender differences, familiarity, and gastronomic perceptions. The aim of this work was to evaluate the perception and acceptance of edible insects by exploring these factors. This study was carried out on higher-education students from universities in Valencia (Spain). The students recognized insects’ high nutritional value, particularly protein content, and had varying levels of knowledge about specific nutritional components. In terms of labeling and marketing, removing health and sustainability benefits from packaging can improve consumer responses. Most respondents prefer clear labeling of insect derivatives, quality certification seals, and complete information about insect content. Students consider marketing and knowledge to be significant influencers of insect consumption. In summary, this text highlights the multifaceted nature of insect consumption acceptability. These insights offer valuable perspectives on insect consumption dynamics.

## 1. Introduction

Novel foods, according to the EU definition (1997), include plant-based, microbial, fungal, algal, and animal-derived products not traditionally consumed in significant quantities by humans. This category also encompasses newly created foods established outside the EU but not traditionally consumed within it [1]. Certain EU regulations, effective since January 2018 (Regulation (EU) 2015/2283), govern the authorization and commercial circulation of novel foods, with additional guidance provided by Commission Implementing Regulation (EU) 2017/2470 [2,3]. Amendments made from 2018 to 2023 detail authorized new foods, specifying names, conditions of use, specifications, and consumer information. Authorization requires confirmation of a product’s pre-1997 consumption history in the EU, consultation, and verification by the Commission of Member States. Safety evaluation by the EFSA is mandatory before marketing authorization [2]. The “Catalogue of Novel Foods”, a guidance document, is the result of ongoing discussions within the Novel Foods Working Group of the EFSA, involving experts from Member States and the European Commission [4].

Since 2018, the number of novel food applications scientifically evaluated by the EFSA has considerably increased due to the new harmonized European regulatory framework. Factors such as provisions that enhance competition and evolving societal needs contribute to this heightened activity [5].

In recent years, edible insects have gained recognition as a more sustainable source of protein compared to other animal-derived proteins. They are being considered a future food and could soon be found in supermarkets and utilized by the food industry as ingredients. In a recently published review, the importance of this novel food was described not only as a source of proteins but also as a source of bioactive compounds that can be a function of diet but also related to insect chemical defense [6].

The consumption of insects (entomophagy) in European diets is not only a growing trend but also constitutes a new food culture, particularly since the EFSA’s 2015 publication on risk assessment related to insect production and consumption. This assessment stressed the need for separate evaluations of biological and chemical hazards, along with data collection due to the insufficient information available [7].

Countries are responsible for regulating their markets, and food agencies play a crucial role in this process. In Spain, the Institutional Commission of the Spanish Agency for Food Safety and Nutrition (AESAN) communicated in 2022 [8] that the marketing of insects could be authorized if they were sold as a novel food or certified as a traditional food from a third country with a consumption history of at least 25 years.

An example of this Is the acceptance of *Locusta migratoria* and dried *Tenebrio molitor* larvae as new foods in 2021, specified in Execution Regulation (EU) 2021/882 of the European Commission [9]. *Acheta domesticus* and *Tenebrio molitor* larvae were authorized for commercialization in frozen, dried, and powdered forms through Regulations 2021/1975 [10], 2022/169 [11], and 2022/188 [12] of the European Commission.

In the context of novel foods, certification and control by food safety organizations are pivotal in ensuring consumer trust and safety [13]. Label information is equally crucial in shaping consumer perceptions of product safety. Additionally, studies emphasize the importance of food hygiene in production. For example, research on European populations highlights that foods of animal origin, primarily meat, eggs, and their derivatives, pose the greatest perceived risk and that rigorous sanitary inspections conducted by competent authorities enhance consumer confidence [14].

Edible insects, as a novel food category, must undergo evaluations to guarantee product safety and quality and alleviate concerns about cultural acceptance, perceived unpleasantness, and doubts regarding safe farming practices [15,16,17]. Academic training and knowledge can influence consumption patterns, and young people, driven by curiosity and a lower perception of risk, may be a target audience for novel food consumption. A comprehensive study examining insect consumption, reasons for acceptance or refusal, and risk perception is essential to understand this emerging trend, with young populations serving as a valuable group for assessing the acceptance of new foods like edible insects.

In this study, a questionnaire was employed to assess the perceptions of students from various health science programs at local universities and evaluate their knowledge of edible insects.

## 2. Materials and Methods

### 2.1. Study Design

An observational, descriptive, cross-sectional study was conducted to collect data on the consumption of insects and the potential factors influencing their acceptance as a new source of alternative protein in a young population sample (university students) from Valencia (Spain). The study participants were either undergraduates or graduates of Human Nutrition and Dietetics, Pharmacy, Gastronomic Science, Food Science, Veterinary programs, and Quality and Food Safety from Valencia (Spain) and who voluntarily agreed to answer the questionnaire. The total number of questionnaires collected was 235, including responses from 165 women and 70 men, corresponding to an age range of 19 to 35 years old. The data collection tool was a questionnaire created based on a review of previous studies and incorporating two validated questionnaires reported by Guiné et al. [16] and Ros-Baró et al. [18].

The final version consisted of 24 questions relating to the potential factors influencing the acceptance of insect consumption, such as cultural influences, gastronomic potential, the sustainability of food systems, economic and commercialization aspects, and nutrition and health information [16,18]. Ten questions had a binary Yes/No response option, and fourteen were Likert scale survey questions. The Likert scale was used to collect the options of “strongly agree”, “agree”, “disagree”, and “strongly disagree”, implying the need to divide them into two groups: positive response (for “strongly agree” and “agree”) and negative response (“strongly disagree” and “disagree”) groups. The questionnaire also included sociodemographic data, such as the respondents’ gender, age, and studies.

### 2.2. Recruiting Participant in the Questionnaire

The questionnaire was created on the Google Forms platform, which is especially suited for online surveys, and was distributed during teaching sessions from 2022 to 2023 (academic years 2021/2022 and 2022/2023). The first screen contained general information about the study. Prior to completing the questionnaire, each participant had to give consent to participate. To ensure the confidentiality of the results obtained, the questionnaires were anonymous, and participants could not be identified.

The study was conducted in compliance with the ethical principles for research involving human beings and the processing of personal data contained in the Declaration of Helsinki and was approved by the Ethics Committee of Research on Humans of the Ethics Commission for Experimental Research of the University of Valencia (cod. 1942475).

### 2.3. Data Analysis

A ten-item questionnaire with a binary scale (Yes/No) and fourteen items each scored on a 5-point Likert scale was used. Yes/No responses were considered nominal and dichotomous categorical variables. Pearson’s Chi-Squared test, which incorporates a non-parametric test to measure the differences between an observed distribution and a theoretical one, allowed the relationship between these dichotomous variables to be analyzed. Statistical analysis of data was carried out using the IBM SPSS Statistic version 23.0 (SPSS, Chicago, IL, USA). The statistical analysis of the results was performed using the Student’s *t*-test for paired samples. Differences between groups were analyzed statistically via ANOVA, followed by the Tukey HDS post hoc test for multiple comparisons. *p* ≤ 0.05 was considered statistically significant.

## 3. Results and Discussion

### 3.1. Preparation of the Questionnaire

The selection of questions was based on validated surveys available in bibliographic sources. In the literature, studies on the perception and acceptance of entomophagy have included models based on a dependent variable such as dietary behavior and independent variables that have an effect on it, so, in the present study, these aspects were taken into account. The independent variables were as follows:

– Neophobia toward food, disgust with insects, and risk assessment of entomophagy are variables that have a negative influence.
– The environmental and nutritional awareness of the participants and their familiarity with entomophagy have a positive influence.
– Sociodemographic variables are also usually included since it is often assumed that these also influence the acceptance of entomophagy; in this case, these variables were gender, age, and the education of the participants.

Because exposure and pleasant taste experiences were recognized as essential elements for enhancing the acceptability of incorporating insects in one’s diet [16], different questions were added. A greater effect was indicated by a complication of emotional elements such as disgust and neophobia, as well as familiar tastes, textures, and settings. Due to the fact that quality certification gives more confidence to consumers and the natural aspect of an insect determines its acceptance, label preferences were also included in the questionnaire.

Culture and tradition were measured through the extent to which edible insects were included or excluded from one’s cultural heritage. Indeed, insect consumption is closely associated with cultural values, religious festivities, local customs, taboos, and traditional knowledge [19,20,21]. Gastronomic potential, including innovation and gourmet cooking, was evaluated due to the fact that certain key subjects can incentivize and influence the improvement of the acceptability of edible insects. Environment and sustainability dimension are matters that consumers are more alert of, making them more prone to change their diets in favor of more sustainable food choices [17,22].

Finally, regarding the dimension of nutritional aspects, four items were included regarding edible insects as sources of high nutritional value [23] and high amounts of proteins, fats, vitamins, and minerals [24] and sources of anti-nutrients, like oxalates and phytic acid [25,26,27,28], related to reducing the bioavailability and/or utilization of nutrients if consumed in large quantities and over a long period of time [28].

### 3.2. Results of the Perception Questionnaire

#### 3.2.1. Dietary Habits

In the literature, different studies about the acceptability of insects as food [29,30,31,32], as ingredients in food products [33,34,35,36,37], as alternatives for meat protein [14,18,38,39], or as insect-based feed [40,41,42] have been reported. In this regard, many studies indicate that upon comparing food before and after being tasted, there is an increase in the intention to eat products containing insect flour as well as a more favorable attitude toward the behavior in accepting these types of food [33]; furthermore, Erhard et al. [37] observed that food neophobia was found to be a strong predictor of willingness to try insect-based foods, whereas food disgust sensitivity had no effect.

**Figure 1:**
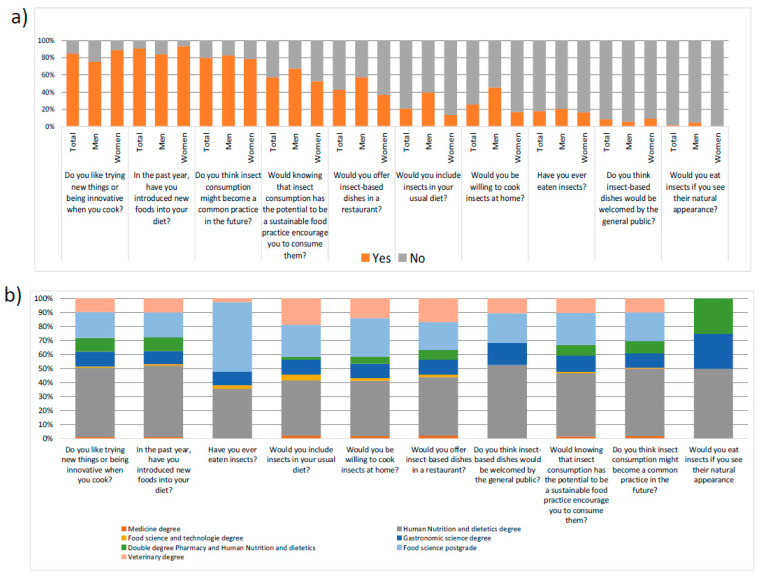
Dietary habits of the respondents according to gender (**a**) and studies (**b**).

Our results regarding dietary habits and the acceptance of insect-based food are reported in Figure 1. Although only 18% of the respondents had tasted insects or insect food products, a favorable attitude toward trying novel food and insects (>84%) accompanied by a positive response (answers of strongly agree and agree) to having been introduced to a new food product in the last year were observed (90%). A positive response was also obtained for the question related to being concerned that “insect consumption will be a future common practice that will be raised up” and consequently “driven towards sustainability consumption”, accounting for 79% and 57% of responders, respectively. This positive acceptance started to decrease when the participants were asked about “offering insect meals in a restaurant” and the “inclusion of insects on a diet daily basis”, with these questions being answered positively by 42% and 21% of responders, respectively (Figure 1a). However, a clear increasing tendency of refusing to incorporate insect food products into dietary habits was observed for aspects related to “cooking insect food”, “introduction of insects daily”, “having tried them”, and “well-acceptance of all consumers or the intentions of eating them by its natural aspect” (with responses ranging from 82% to 97%). Notably, positive responses to many questions and concretely with respect to “to include insects in diet” were related to those pursuing degrees in human nutrition and dietetics (40%) or postgraduate students in food science (23%) (Figure 1b).

Another aspect to highlight in this block is gender, especially regarding the question about trying new foods (Figure 1a). Women reported a greater intention to try new food compared to men (89% women vs. 75% men); however, regarding who would be willing to “include insects in their diets or cooking insects at home” or would not be averse to “offering insects in a restaurant”, the intention was higher for men (57% of men vs. 37% of women and 46% of men vs. 17% of women, respectively), revealing that men have a greater acceptance of eating insects.

#### 3.2.2. Perception of Acceptability of Eating Insects

**Figure 2:**
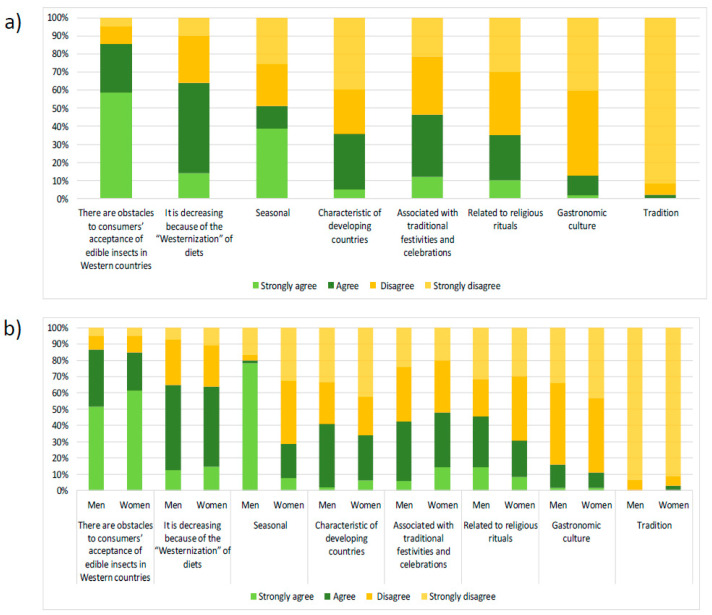
Reasons for eating insects arranged by total respondents (**a**) and gender (**b**).

Studies on insect consumption acceptability highlight the role of familiarity in reducing food disgust. Our results, as shown in Figure 2a, reveal that Valencian university students primarily associated their perception with a decrease in the “tendency in occidental diet” (64%). This suggests a willingness to adopt a more open diet. Other factors influencing perception included the “seasonal” nature, commonality in countries concerned about future food perspectives, association with festivities and religious rituals, and adherence to “tradition”, with agreement levels ranging from 8% to 47% for positive perspectives (answers of agree and strongly agree). However, from the negative perspective (for answers of disagree and strongly disagree), the greatest negative perspective on insect consumption was reported for being something “traditional” (98%), while the lowest negative perspective was for the factor of “seasonal” perception (49%). Notably, “tradition” (92%) and “culture” (87%) were discarded as the reasons associated with insect consumption by populations (Figure 2a).

A last insight of this block of questions was that insect consumption was associated with “developed countries” (36%) where there are difficulties regarding insect consumption (86%) and their consumption has decreased due to the “Westernization” of diets (58%). Regarding gender, it was observed that men consider the consumption of insects to be “seasonal”; in fact, 79% of male responders considered this the main reason for eating insects (Figure 2b).

#### 3.2.3. Gastronomic Perception of Insects

**Figure 3:**
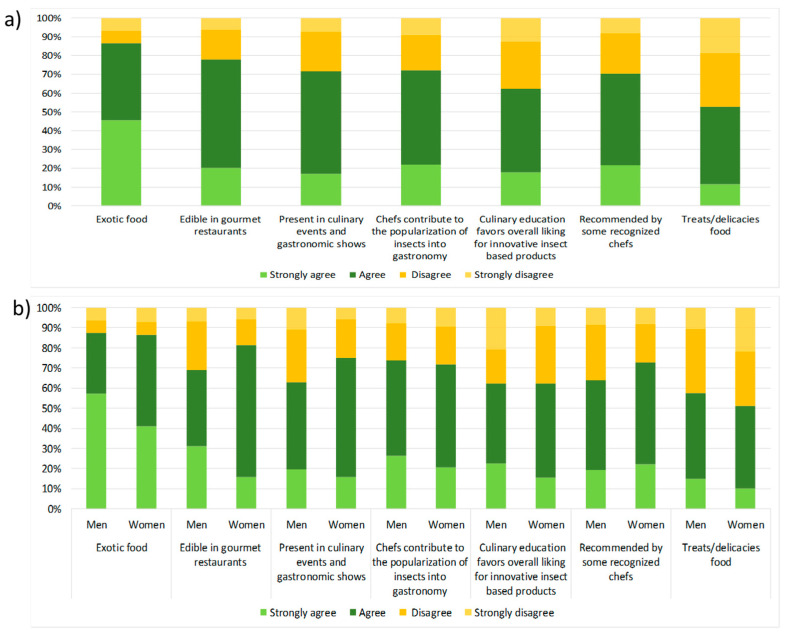
Gastronomic perception of insects by total respondents (**a**) and by gender (**b**).

The responses related to the perception of insect consumption and conceived from a gastronomic point of view include aspects of “characteristics in food” or because food marks “an special occasion”. The questionnaire included an evaluation of gastronomic situations wherein insect consumption could be more frequent (Figure 3). The following factors were considered: “exotic food”, “treats/delicacies food”, “edible in gourmet restaurants”, “present in culinary events and gastronomic shows”, “recommended by some recognized chefs”, “chefs contribute to the popularization of insects into gastronomy”, and “culinary education favours overall liking for innovative insect based products” (Figure 3).

The results reveal that out of all factors, a total of 82% of the respondents considered the gastronomic perception of insects as edible to be associated with “exotic food”, while 78% considered it to be associated with being “available in gourmet restaurants”, and it has been the chefs who have contributed to the popularization of this food in culinary events and gastronomic shows (72%) (Figure 3a). On the contrary, the respondents revealed that gastronomic perception is not associated at all with the fact that a meal can be considered “treat food” (candies) or part of “culinary nutritional education”, and the fact of it being “available in culinary events” contributes to its popularization, with the values observed ranging from 52% to 71%. The two factors about chefs were perceived to be less of a positive motivation to consume edible insects (72%), corresponding to an equal frequency. However, for women, the impact of “insects consume being recommended by some recognized chefs” (72%) was higher than it was for men (62%) (Figure 3b).

#### 3.2.4. Knowledge of Insect Safety Quality and the Effect of Insect Consumption on Health

Regarding the acceptance or rejection of insects’ consumption, it is relevant to determine the types of degrees the respondents have obtained (education) and their levels of knowledge and awareness regarding sustainability issues, as these factors can contribute to their perception of insect consumption. Although the literature contains different methodologies related to this topic, such as the Food Neophobia Scale [43] (to highlight that it is one of the most used), these methods do not apply specifically to edible insects and do not cover the range of domains that were included in our questionnaire. The dimension considered was health knowledge, which is essentially related to the risks associated with the consumption of insects and knowledge of their quality with respect to safety. Consumers tend to have decreased trust for foods that they are not familiar with and consider them to pose a higher level of risk than other foods, especially when higher risk is involved.

**Figure 4:**
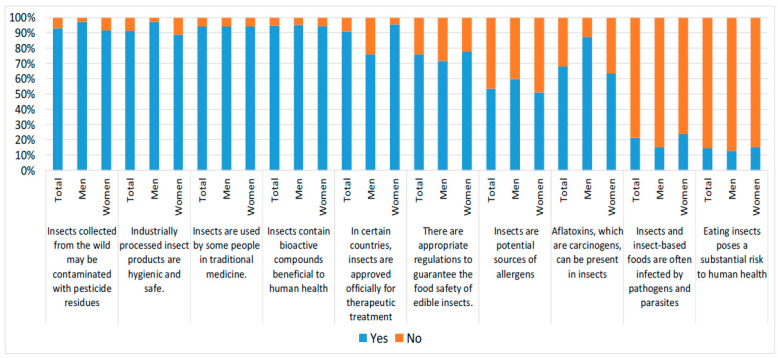
Knowledge of the safety and effects of insect consumption on health.

Although the perception of risks is usually high regarding insect consumption, in our study, the university students declared that, regarding aspects related to health, it is highly possible that “insects collected from forests may be contaminated with pesticide residues” (93%); accordingly, 91% of the reported that “industrially processed insect products are hygienic and safe” (Figure 4). Among these respondents, it was revealed that they understood that “insects are used by some people in traditional medicine” (94%). In the same tendency of concern for and perception of the benefits and healthy aspects offered by insect consumption, there was a clear positive response to the fact that “insects contain bioactive compounds beneficial for human health” (95%), as well as to the fact its use in some cultures for therapeutic treatment is officially approved (91%), or that “eating insects does not pose a substantial risk to human health” (86%) and that they are not infected by pathogens or parasites (79%) (Figure 4).

In this section, it is also important to remark that there is uncertainty and there are unknown concepts related to the health benefits of insect consumption, for example, the presence of contaminants such as aflatoxins in insects (81%) and the possibility of insects being a potential source of allergens (67%) (Figure 4).

To summarize, the consumption of insects is perceived as safe, including all good practices followed in their production and transformation, just as occurs with other types of food. However, if the insects are collected from the wild/forests, the responders indicate that they may be contaminated with pesticide residues, contributing to the risk perception of insect consumption (Figure 4).

Regarding regulations for insects’ consumption, the students ignored whether there was European legislation, and the item related to this dimension was answered positively by 33% (76 students), indicating that “there are appropriate regulations to guarantee the food safety of edible insects”; however, 56% (131 students) of respondents ignored whether there are regulations to guarantee the food safety of edible insects. This indicates to us that the students were not informed about the recent regulation established by the European Commission [8,9,10,11].

#### 3.2.5. Knowledge of the Nutritional and Health Contributions of Insects

**Figure 5:**
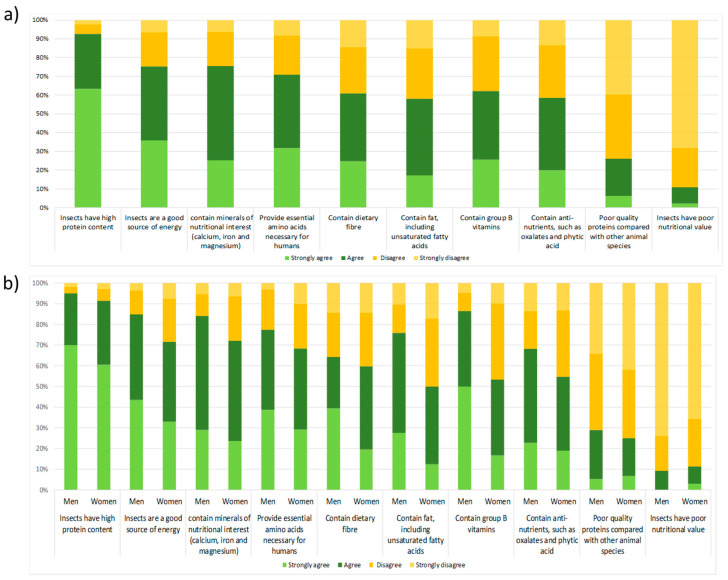
Knowledge of the nutritional contribution of insects arranged by total respondents (**a**) and by gender (**b**).

The questionnaire considered the dimension of nutritional aspects by analyzing them in two directions: (i) through the nutritional contribution and (ii) through the education profiles of the responders. It was confirmed that the nutritional quality of the consumption of insects was the most known aspect. The majority of the students indicated that insects have high nutritional value because they provide high protein content (91%) and are a good source of energy (75%). When they were asked about their specific knowledge with respect to providing specific nutritional components, the following order was attained (from strongly agree to strongly disagree): providing nutritional minerals, essential amino acids, dietetic fiber, fatty acids, vitamin B group compounds, phitic acid and oxalates, poor quality of protein content, and poor nutritional content (Figure 5); the responses ranged from 26% to 76%.

**Figure 6:**
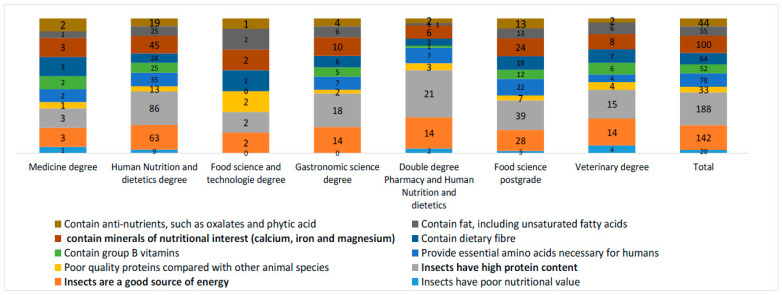
Knowledge of the nutritional contribution of insects (in bold: the most known option).

Regarding the educational profiles or degrees, it was observed that the respondents with human nutrition and dietetic degrees were convinced that insects are a good source of energy and have high protein content, along with the respondents with double degrees in pharmacy and human nutrition and dietetics and food science postgraduate students, with both groups being considered to have the highest levels of knowledge related to food safety (Figure 6). Nevertheless, there was also a general perception collected from the respondents that although their degrees were less related to food perception, both factors of good protein and energy sources were also the ones with the highest degree of importance.

#### 3.2.6. Influence of Marketing and Labeling Preferences on Purchase and Consumption

There are studies on the effect of the visual appearance of a real insect on product packaging, indicating that it can trigger a disgust-based food rejection [44], while others report an increased willingness to eat products when the insect ingredients are less visible compared to products that contain unprocessed insect ingredients [45,46]. It has been concluded that removing the image of an insect from product packaging can have a beneficial effect on perceived disgust; removing any references to insects can be perceived of as a deceptive strategy and therefore lead to negative consumer reactions [47,48].

**Figure 7:**
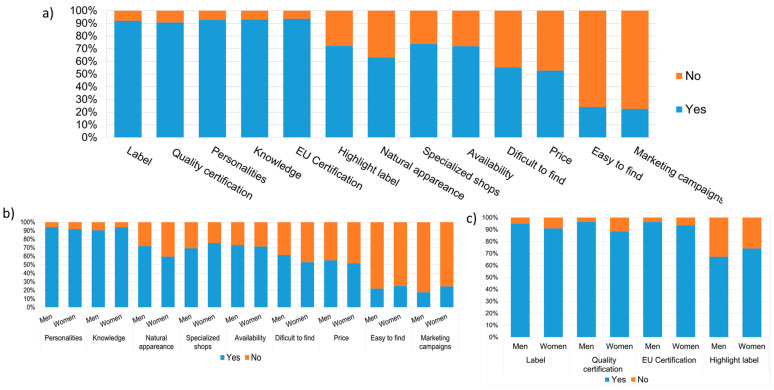
Influence of marketing and label according to total respondents (**a**) and gender ((**b**) marketing and (**c**) label) on purchase and consumption.

However, our results, reported in Figure 7, suggest that regarding the preferences of information appearing on the label, our responders found it highly preferable to indicate the presence of insect derivatives as ingredients (92%), followed by the presence of a quality certification seal (91%), and they also preferred the natural appearance on the product package (63%), as well as its being correctly labelled, fully indicating the information about insect content (92%). Regarding the aspect of certification, the students’ perceived a product to be safe if the label indicated information about a certification complying with European regulations via an identification mark and a health mark (94%) and perceived greater product safety if a product has a quality certification seal (91%).

Related to food packaging and food labelling, marketing enters as an important factor. In fact, marketing has implications for the communication of product benefits and qualities. However, marketers have to be careful when attempting to promote product healthiness and sustainability, especially through product packaging. Pozharliev et al. [44] showed that removing the focus on health- and sustainability-related benefits from product packaging improves the implicit, self-reported, intentional, and behavioral responses to insect-based food products of first-time users. Thus, in our questionnaire, it was included to evaluate the perception of the influence of marketing campaigns on consumption. The results revealed that 134 students (77%) considered marketing for insect consumption to be crucial; however, many of them declared that “The level of knowledge influences the willingness to purchase insect food” (93%).

## 4. Conclusions

The acceptance of introducing insects as new foods into the diets of Spanish university students was assessed heroin based on a perception questionnaire. The results showed that while a small proportion of participants had tried insects or insect-based food products and while a few were willing to include them in their regular diet, there was a positive attitude toward consuming them in the future, especially due to sustainability. The main reason for the low consumption of insects was the traditional and cultural aspects typical of Western countries, making them seem exotic in terms of gastronomy. Regarding nutrition and food safety, most participants believed in the nutritional and health benefits of insects and trusted the hygiene standards of the food industry over direct field collection. When it comes to consumer information, this study found that product acceptance could be improved by using certifications from food control and safety organizations to ensure product quality and safety. While gender differences are often observed in studies related to food risk perception, this study did not find substantial differences between males and females, except for minor variations in dietary habits and some considerations about the seasonality and nutritional components of entomophagy.

The availability of edible insects to consumers underscores the complexities and intricacies of our relationship with food, culture, nature, and sustainability. Although struggles with these questions occur, the acceptance of edible insects reflects a shift toward a more environmentally conscious and ethically responsible approach to food, challenging long-standing norms and encouraging us to think more deeply about our place in the natural world.

## Data Availability

Data are contained within the article.
